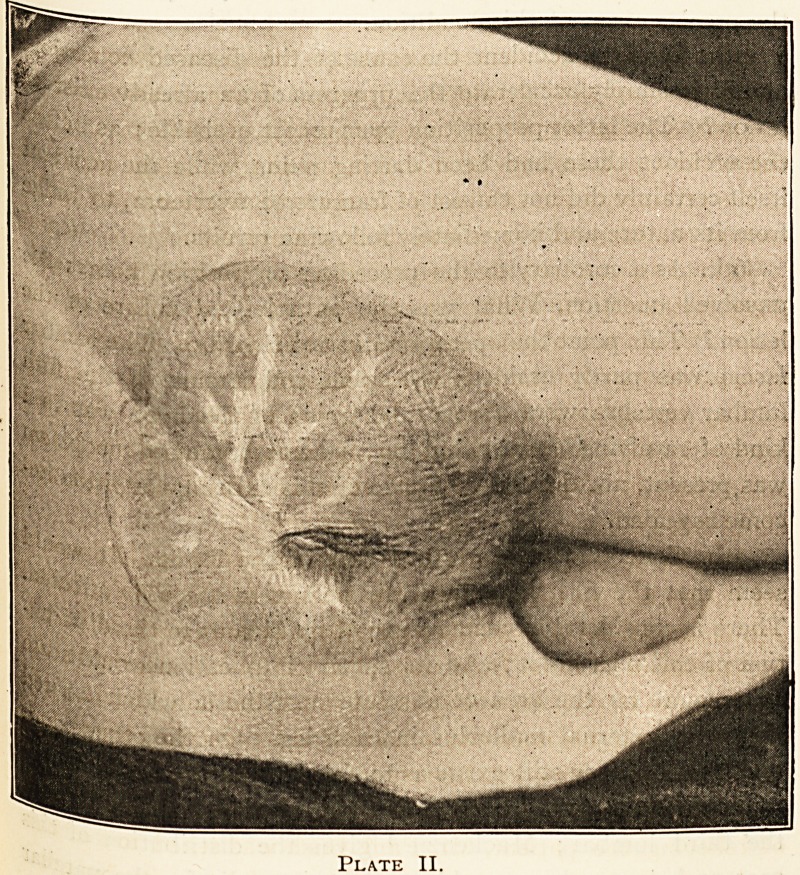# Lesion of the Cauda Equina: Operation—Relief of Symptoms

**Published:** 1893-09

**Authors:** J. E. Shaw, J. Paul Bush

**Affiliations:** Physician to the Bristol Royal Infirmary; Surgeon to the Bristol Royal Infirmary


### LESION OF THE CAUDA EQUINA: OPERATION—RELIEF OF SYMPTOMS.

BY

J. E. Shaw, M.B. Edin.,

*Physician to the Bristol Royal Infirmary,*

AND

J. Paul Bush, M.R.C.S. Eng.,

*Surgeon to the Bristol Royal Infirmary.*

---

### OBSERVATIONS BY DR. SHAW.

As in this country comparatively few cases of lesion of the cauda equina have been observed and subjected to treatment by operation, it seems desirable to place this case upon record, although perhaps it does not distinctly add anything to our rather imperfect knowledge of the distribution of the lumbar and sacral nerve-roots. The symptoms and diagnosis of lesions of the cauda equina have been admirably discussed by Thorburn [1], and again more recently by Allen Starr [2], who has quoted several cases which have been recorded since Thorburn's work was published. It would be superfluous, therefore, to discuss these questions here; so, reserving until later some comments upon the case, I pass at once to the notes describing the patient's condition.

W. D., male, æt. 30, bootmaker, was admitted to the Bristol Royal Infirmary on February 10th, 1893, complaining of weakness, pain and loss of sensation in the legs, and loss of control over the functions of micturition and defæcation.

*Previous History.*—Patient suffered from rheumatic fever twice in childhood. At the age of 12 he had an attack of typhus fever. He has never been strong; but enjoyed fair health from 16 to 20 years of age. For the last 14 years he has been employed in the bootmaking trade.

*Family History.*—Father, æt. 58, and mother, æt. 57, are living and healthy. A brother and a sister living and healthy. Five others died in infancy.

[1] *The Surgery of the Spinal Cord*, 1889. [2] *Amer. Journ. Med. Sci.*, July, 1892.

*History of Present Illness.*—Eleven years ago, while carrying a banner in a procession, patient became engaged in a struggle with another man, and sustained a fall, wrenching the lower part of his back. He dates his illness from that time; but says also that for two or three years before, he had felt slight dull pains, more or less constant, in the popliteal spaces and outer sides of legs and heels. A few days after the accident, he began to feel gnawing pain in the back of the thighs, constant, but with exacerbations at intervals. This became worse, and was accompanied by pain and tenderness in the lower part of the spine. About two months after, he began to notice that his legs were getting weak, and this weakness has been gradually increasing up to the present time; he has become less and less able to walk any long distance, and for the last two years has been unable to walk without the support of a stick. About the same time he began to notice the anæsthesia, which, in his belief, began in the backs of the thighs and spread downwards; he is not certain when the anæsthesia around the anus first appeared. About three months after the accident he began to lose control over the rectum; and about that time he entered a hospital in a neighbouring city, where for five weeks he was treated for "rheumatic sciatica," but left worse than upon admission. Control over the rectum gradually grew less and less until four years ago, when, while attending the Bristol General Hospital as an out-patient, although his bowels were acting two or three times a day, it was found that the rectum contained a quantity of scybala, which were removed. Since then he has been in the habit of using a soap-and-water enema every second day, without which his bowels are not relieved, unless he should take a brisk purgative, when incontinence of fæces results. About eight months from the commencement of his illness he began to find that he had to micturate more frequently; this frequency gradually increased, and after a time micturition became painful, and when his bladder was full urine ran away involuntarily. At that time he first noticed that his urine was turbid, had an ammoniacal smell, and deposited a sediment on standing. Gradually he began to lose altogether the power of voluntary micturition, and for the last four years he has passed a No. 10

catheter several times a day. Until about two years ago the urine used to overflow if he did not pass the catheter every few hours, but now he can retain it for about eight hours. Of late years he has had painful spasmodic jerkings of the hamstring muscles. Since the commencement of his illness he has had nocturnal erections and emissions, attended with sensation, pretty regularly about once a month; and during the last few months they have occasionally occurred when he was awake, attended also with sensation. Two years ago a "corn" appeared on the ball of the right great toe, which ulcerated, and has never since healed. About that time also, while sitting over a pan of very hot water, in order to get an action of the bowels, he sat into the water, and, being completely anæsthetic, scalded himself severely round the anus and over the sacrum without being conscious of the occurrence at the time.

Condition on Admission.—Patient is pallid and unhealthy looking, although not emaciated. He usually lies upon his right side with his legs drawn up.

*Urine.*—Alkaline; sp. gr. 1017; slightly turbid; contains some albumen, and forms a slight deposit of pus.

*Respiratory System.*—Normal.

*Circulatory System.*—Pulse regular, 75; moderately full. No cardiac murmur.

*Digestive System.*—Appetite good. Bowels act only after enemata. Tongue has a slight whitish fur.

*Nervous System.*—The fourth lumbar spine (or fifth, it was uncertain which, for reasons which appeared at the time of the operation) is somewhat prominent, and at that point, and for a short distance below, there is tenderness on percussion and on the application of a hot test-tube or sponge. *Reflexes:* Right knee-jerk, normal; left, slightly exaggerated. Neither patellar nor ankle clonus obtainable. Plantar reflex absent on both sides: the cremasteric present, and the abdominal and epigastric exaggerated.

*Electrical Reactions.*—The Faradaic irritability of the hamstring muscles, the peronei, the calf, and tibial muscles is much diminished, especially upon the left side, but not completely absent; the irritability of the right glutei is diminished slightly, and of the left glutei somewhat more. The galvanic reactions are as follows:

| | | |
|---|---|---|
| Quadriceps Extensor | KCC > ACC | AOC > KOC |
| Hamstring Muscles | ACC > KCC | AOC > KOC |
| Calf Muscles ... ... | ACC > KCC | KOC > AOC |
| Peronei ... ... ... | ACC > KCC | KOC > AOC |
| Glutei ... ... ... | ACC > KCC | KOC > AOC |

His legs are weak, and he is unable to walk more than a short distance. In walking he scarcely lifts the feet from the ground; and the left leg is weaker than the right. Adduction and abduction of the thigh are performed well, as are flexion of the thigh and extension of the knee; but all movements performed by the hamstrings and by the leg and foot muscles are weak, and extension of the hip-joint is somewhat weakened, especially on the left side. The hamstring muscles and all the muscles below the knee are considerably wasted; the feet are arched in the sole and slightly inverted; the toes are extended

PLATE I.

at the metatarso-phalangeal joints and flexed at the interphalangeal. On the ball of the right great toe there is a callosity with a narrow ulcer extending inwards about three-fifths of an inch, but not reaching down to the bone. (*Vide* Plate I.)

Fibrillary tremors of the affected muscles may be seen at times.

The skin over the coccyx and round the anus is completely anæsthetic to all forms of sensory stimulation. Anæsthesia of a less intense degree exists over the nates, backs and inner sides of the thighs, popliteal spaces, calves, lower parts of the fronts of the legs, to a slight degree on the outer side of the left leg, and the entire feet except upon the inner side of each foot.

(*Vide* diagrams, where depth of shading denotes degree of anæsthesia.) There is a specially anæsthetic area above and below the external malleolus on each side. The area of skin supplied by the patellar plexus is very slightly anæsthetic. Sensibility to touch, heat, and pain is about equally affected; but the skin of the penis and scrotum is sensitive to touch, but not to pain (indicated in diagram by shading being in dots instead of in lines). Urethra is completely anæsthetic, but testicular sensibility is present. If patient allows the bladder to become very full, a small amount of urine runs away. Postural sensation in the legs is much impaired, but not annihilated. The tone of the sphincter ani is very feeble (the patency of the anus is apparent in the representation in Plate II.); the mucous membrane of the rectum is anæsthetic. His bowels do not act without an enema, which is administered with great difficulty owing to the exceedingly imperfect capability of the sphincter ani to retain the fluid. When his bladder or rectum is full, patient says that he is conscious of the fact, but describes it as "not the natural feeling, but a tight feeling." Round the anus and over the coccyx is the cicatrix of the scald which he sustained two years ago, as above described; it is roundish in shape and about five inches in diameter. (*Vide* Plate II.)

Patient suffers intensely from darting pains in the back of the thighs and in the legs, so as to require for their relief hypodermic injections of morphia every three or four hours. Lately he has begun to suffer from pain of a less severe degree in the front of the thighs, especially in the inner and outer parts. He considers that his muscular weakness is increasing slowly, and the pain is becoming much more intense in its severity.

As no other line of treatment afforded a reasonable prospect of relieving the patient, Mr. Paul Bush was asked to see him for the purpose of arranging to perform laminectomy. No great hope of amendment was held out as likely to be derived from surgical interference; but the patient's sufferings being so intense, he desired to be operated upon, and for that purpose was transferred to Mr. Bush's charge on March 16th.

Summarising the points to be observed in this case, it may be noted that:—

1st. Pain, paralysis (motor), and anæsthesia was the order of development of the nerve symptoms here, as in all these cases.

2nd. Contrary to what one might expect upon *a priori* grounds, the state of the sphincters of the bladder and rectum is such that retention, and not incontinence, of their contents obtains.

3rd. Notwithstanding that the patient is a very intelligent

Plate II.

man, we may be sure that his statement that the anæsthesia began in the backs of the thighs and spread upwards and downwards is incorrect. It seems to be an invariable rule that in cases of this nature, when any one of the sacral nerve roots is affected, those below it are also included in that affection. The concentric distribution in respect of cutaneous sensibility of the sacral nerve roots [1] renders it, therefore, practically certain that this patient's coccygeal region was anæsthetic at the same time as, if not before, the backs of the thighs.

4th. The case is apparently unique in the fact that the penis and scrotum were analgesic but *not* anæsthetic. Repeated examinations upon this point were made so as to be certain of the fact. It is difficult to offer any satisfactory suggestion as to the precise cause of this condition.

5th. Was the accident the cause of the diseased condition, or did it simply accelerate the progress of an already existing lesion? The latter proposition seems most probable; as before the accident there had been darting pains, while the accident itself certainly did not consist of fracture of a vertebra, to judge from its nature and immediately following results.

6th. As a corollary to the preceding proposition comes the unsolved question, What was the pathological nature of the lesion? This point the operation did not clear up. The lumbar fascia was partly ossified; the spine and laminæ of the fifth lumbar vertebra were absent, and much of the bone was in a kind of rarefying osteitis, but no pus nor organised neoplasm was present, nor did the *vera causa* of this existing condition become revealed.

7th. Concerning the upward limit of the lesion. It would seem that the fifth lumbar nerve roots were specially affected. There is considerable concurrence of opinion as to the distribution of this nerve root; and its special implication would seem to account for the area of absolute anæsthesia which existed above the external malleolus in each leg. On the other hand, much uncertainty still exists as to the distribution of the fourth lumbar nerve roots. Starr [2] does not separately define it from the third lumbar; Mackenzie [3] gives the distribution of this root as being such as would account *inter alia* for the patellar plexus being anæsthetic in this case. It is practically certain also that in this case the third lumbar nerve roots were affected to the degree of producing some pain and anæsthesia, but scarcely of distinct motor paralysis; the motor power and

[1] *Vide* diagram, *Amer. Journ. Med. Sci.*, July, 1892, p. 29.

[2] *Loc. cit.* [3] *Journ. Path. and Bact.*, No. III., p. 343.

electrical reactions of the extensor muscles of the thighs did not recognisably deviate from the normal. Again, the recent supervention of darting pains in the region of the second lumbar nerve roots showed that they were becoming involved in the upward extension of the diseased condition, and induced both the patient and surgeon to agree to an operation being performed. The first lumbar nerve roots, and all those still higher, were quite unaffected.

8th. The electrical reactions gave in a varying, but not complete, degree the "reaction of degeneration" in all the muscles moving the thighs, legs, and feet, except the quadriceps extensor femoris and sartorius on each side, showing thus the "peripheral" type of the lesion.

9th. It is a matter almost of wonder that the trophic functions of the affected parts were not more disturbed over a course of years. The scald in the coccygeal area appears to have healed readily; the flaps made at the operation showed but little less than normal vitality; the toe-nails show no signs of defective nutrition. The perforating ulcer alone gave proof of trophic defect, and that healed immediately after the operation.

### OBSERVATIONS BY MR. BUSH.

This case did not offer much promise of cure. As the pressure symptoms were of so many years standing, one felt that in all probability some or most of the nerves implicated in the cauda equina were very much disorganised.

On March 16th the patient was anæsthetised with ether, and an incision was made about nine inches long in a vertical direction, extending from two inches above the level of the crest of the ilium to the fold between the buttocks, and over the spines of the vertebræ. It was found necessary, so as to give more room, to make a second incision transversely about four inches long, and meeting the vertical incision about three inches below its upper extremity. The muscles and fasciæ on each side of the spinous processes were then dissected off, and a swelling exposed situated in the middle line: this was freely incised, when a rounded cavity, covered in with a thin shell of bone some three inches across, came into view. The spine of the fourth lumbar vertebra was found to be pushed backwards: this was removed, together with the laminæ on either side, by means of the bone forceps. The fifth lumbar spine and laminæ appeared to have been entirely absorbed; the cavity, which was evidently the dilated spinal canal, contained a solid mass, which was removed, some hemorrhage occurring. The nerves forming the cauda equina at this position could not be clearly defined, as they were pressed forward against the posterior surface of the bodies of the vertebræ, and covered over by strong fibrous tissue. In removing this mass it was torn into several pieces, one of these fragments being as large as an ordinary orange. The soft parts were brought together with numerous silkworm-gut sutures, and a large drainage tube inserted. There was a good deal of bloody oozing for the first twenty-four hours. The day after the operation the patient said he was feeling better, and that the pains in the legs were very much less severe; he was placed on a water-bed, as there was a great tendency in the flaps to slough on the slightest continued pressure on any one point.

March 20th.—Wound dressed each day. Patient complained to-day of some return of the old starting and pains in the legs.

March 21st.—The upper part of the wound is healed; the pain is much less. The patient has now only one hypodermic injection of morphia in the twenty-four hours, instead of one every three or four hours as before the operation. There is some returning sensation (tactile) over the upper of the anæsthetic areas.

March 25th.—Sutures removed; tube shortened. There is a free discharge of cerebro-spinal fluid.

April 10th.—The last day or two there has been swelling and pain over the lower portion of the wound. Half ounce of pus let out to-day.

April 20th.—Patient allowed up on the couch.

April 29th.—Patient goes into the garden each day. Wound still discharging pus slightly. No cerebro-spinal fluid since 8th instant.

May 8th.—Patient is looking a different man since the operation. Eats his food well. Morphia discontinued altogether. Enemata are now retained till the required action of the bowels takes place. The condition of the affected muscles of the legs remains unchanged as regards power of movement and electrical reactions. The pain, which formerly was constant, is now felt only at times, and then much less severely. The degree of anæsthesia is diminished, there being now no area of *complete* anæsthesia; and the original areas of impaired sensation are now improved.

June 4th.—There is now some slight improvement in the power of movement in the paretic muscles of the legs; but there does not appear to be any decided alteration in the electrical reactions. Patient says that he can feel pain in the urethra when the catheter is passed; and the skin of the scrotum and penis has become again sensitive to pain as well as to touch.

June 30th.—Patient is in much the same condition, excepting that during the last few days some improvement has been observed in the electrical reactions. The mass from the spinal canal shows, under the microscope, well-organised blood-clot with some fibrous tissue, but no other distinctive structure.

## Figures and Tables

**Plate I f1:**
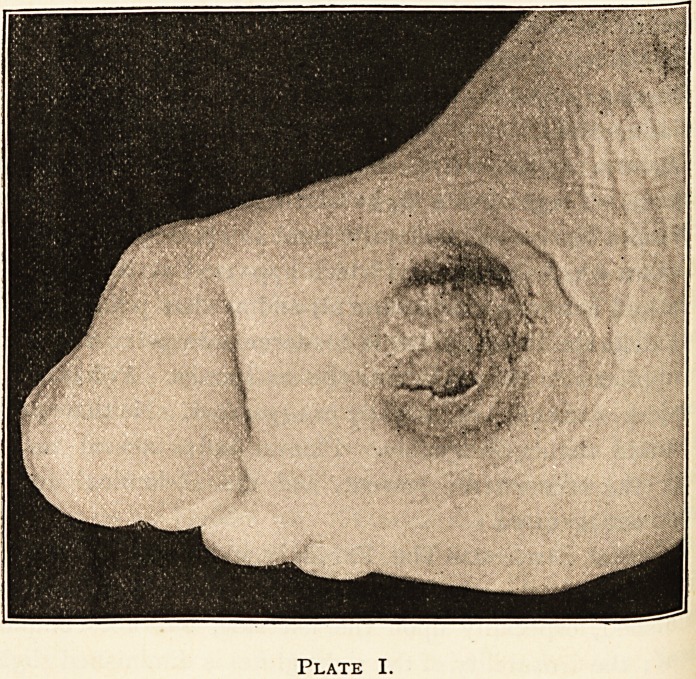


**Figure f2:**
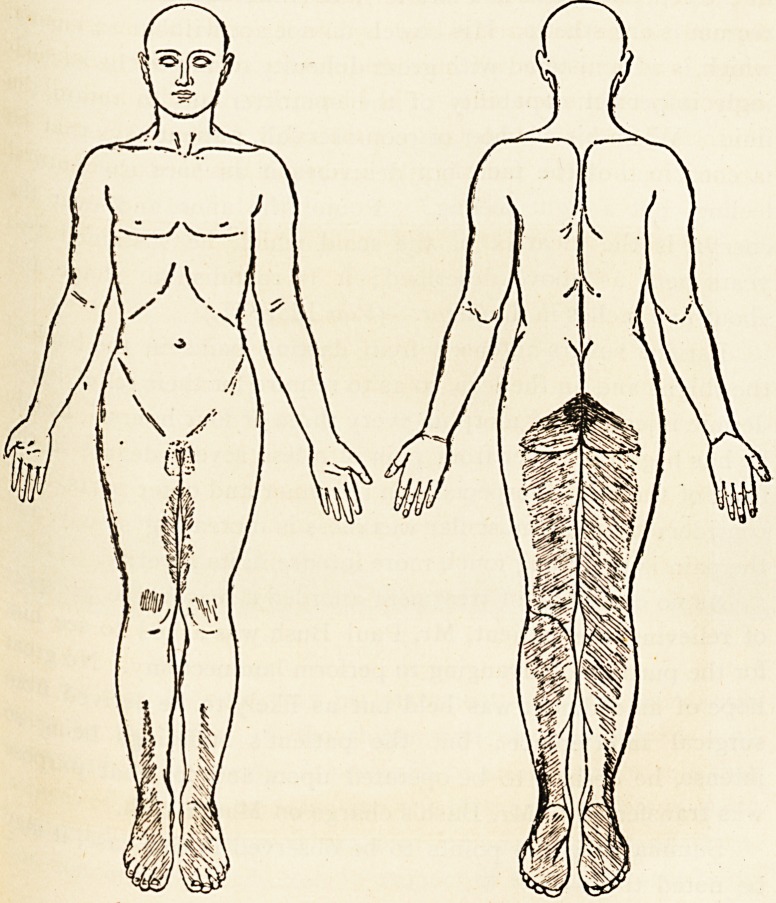


**Plate II f3:**